# The damping of terahertz acoustic modes in aqueous nanoparticle suspensions

**DOI:** 10.1038/s41598-021-99503-6

**Published:** 2021-10-11

**Authors:** Alessio De Francesco, Luisa Scaccia, Ferdinando Formisano, Eleonora Guarini, Ubaldo Bafile, Marco Maccarini, Yugang Zhang, Dmytro Nykypanchuck, Ahmet Alatas, Alessandro Cunsolo

**Affiliations:** 1 CNR-IOM & INSIDE@ILL c/o Operative Group in Grenoble (OGG), 38042 Grenoble, France; 2 Institut Laue-Langevin (ILL), 38042 Grenoble, France; 3 Dipartimento di Economia e Diritto, Università di Macerata, Via Crescimbeni 20, 62100 Macerata, Italy; 4 Dipartimento di Fisica e Astronomia, Università di Firenze, via G. Sansone 1, 50019 Sesto Fiorentino, Italy; 5 Consiglio Nazionale delle Ricerche, Istituto di Fisica Applicata “Nello Carrara”, via Madonna del Piano 10, 50019 Sesto Fiorentino, Italy; 6 Laboratoire TIMC/IMAG UMR CNRS 5525, Université Grenoble-Alpes, 38000 Grenoble, France; 7 Brookhaven National Laboratory, Center for Functional Nanomaterials, P.O. Box 5000, Upton, 11973 NY USA; 8 Argonne National Laboratory, Advanced Photon Source, P.O. Box 5000, Upton, 11973 NY USA; 9 Department of Physics, University of Wisconsin at Madison, 1150 University Avenue, Madison, WI USA

**Keywords:** Materials science, Nanoscale materials

## Abstract

In this work, we investigate the possibility of controlling the acoustic damping in a liquid when nanoparticles are suspended in it. To shed light on this topic, we performed Inelastic X-Ray Scattering (*IXS*) measurements of the terahertz collective dynamics of aqueous suspensions of nanospheres of various materials, size, and relative concentration, either charged or neutral. A Bayesian analysis of measured spectra indicates that the damping of the two acoustic modes of water increases upon nanoparticle immersion. This effect seems particularly pronounced for the longitudinal acoustic mode, which, whenever visible at all, rapidly damps off when increasing the exchanged wavevector. Results also indicate that the observed effect strongly depends on the material the immersed nanoparticles are made of.

## Introduction

The study of terahertz dynamics of density fluctuations in monatomic, molecular and glass-forming liquids has represented a vivid field of research since the early 1960s. As a result of an intensive theoretical, experimental, and computational scrutiny, this field has nowadays reached full maturity and an increasing interest is being attracted by more complex systems whose dynamic response is hardly formalized by existing theories. Some of these systems are likely to bring a true paradigm shift in this field. In fact, their complex nanoscale structure naturally lends itself to high-level design and manipulation. This potentially changes the very role of the experimenter from a mere observer of physical phenomena occurring in “natural” materials to an architect of artificial and fully tailored devices potentially displaying unusual properties, or enhanced functionalities. Along this route, a growing attention is being directed to the control of acoustic propagation in complex materials via the design of the mesoscale structure. This endeavor appears especially relevant at mesoscopic scales, where phonon excitations are the leading carriers of heat flow in insulators and their control becomes critical to implement heat flow management based upon structural design^1^. A natural pathway to accomplish this goal rests on the use of ordered composite devices, in which the mass and elastic constant distributions have periodicity designed to interfere with sound waves causing them to slow down, deviate or getting trapped in propagation gaps. While in the so-called phononic crystals^2^ acoustic propagation is hampered by multiple Bragg reflections at the interfaces between two periodically-arranged materials, in acoustic metamaterials sound waves exchange energy with nanostuctures acting as local resonators^3^ whose spatial arrangement is not necessarily periodic. In this perspective, one might consider the possibility to implement acoustic manipulation in composite systems lacking large-scale ordering, like fluids. Thus far this endeavor has been primarily held back by the persuasion that acoustic propagation can be more efficiently manipulated by solid-state nano-architectures, while taking advantage of both the large-scale ordering and the higher rigidity of the system. However, growing experimental evidence indicates that the distinction between liquid and solid response becomes more elusive at picosecond and nanometer scales, as revealed, for instance, by the onset of a shear wave propagation in the spectrum of density fluctuations of several liquids^4–11^. Furthermore, the inherent disorder of the liquid-state structure yields in itself a leading contribution to the damping of terahertz acoustic propagation^12,13^, which might inspire attempts to manipulate acoustic propagation by moving a partially ordered system across an order–disorder transition^14^. As an alternative pathway, one can control terahertz sound damping via the inclusion of heterogeneities in a fluid, as, for instance, floating nano-objects. In this case, the elastic inhomogeneity, i.e. the mismatch of elastic properties between floating colloids and hosting liquid can hinder the propagation of sound waves throughout the system, thus possibly decreasing their lifetime. Along this line, recent works^15–19^ demonstrated that the dispersion of nanoparticles (NPs) can enhance the damping of the terahertz collective modes probed by Inelastic X-ray Scattering (*IXS*)^20^ or Neutron Scattering (*INS*)^21^ measurements.

Although the understanding of this damping mechanism is relevant to the manipulation of sound propagation (heat flow) through non-crystalline composite materials and, ultimately to the development of a new class of thermal devices, *IXS* and *INS* works on this topic are still sporadic. This deficit partly owes to the difficult interpretation of the often featureless scattering signal from disordered complex systems, as well as to the lack of a theory of the spectrum of density fluctuations from composite and nanostructured materials. Some challenges in the lineshape modeling can be addressed by the use of Bayesian inference^22,23^, as demonstrated by previous works in which Bayesian methods were used to determine, e.g. the number of exponential terms determining the time-decay of a measured time correlation function^17^, or spectral modes contributing to the inelastic scattering signal^15–19^. In this work, Bayesian inference is used to elucidate the effect that NPs in aqueous suspensions have on the terahertz dynamics of water. In particular, after our recent study devoted to suspensions of Au in water^24^, we consider the case of vitreous silica nanoparticles ($\hbox {vSiO}_{{2}}$-NP) of various size, relative concentration, and electrostatic charge, one of the goals of this work being to explore if and to what extent such parameters may affect the *IXS* spectral shapes. The *IXS* spectra of the Au-NP suspensions and pure water were modeled by the sum of Damped Harmonic Oscillator (DHO) profiles, plus a Lorentzian and a $\delta$-function terms, accounting for the inelastic, quasielastic, and elastic portions of the spectra, respectively. Conversely the statistically most grounded model best-fitting of the $\hbox {vSiO}_{{2}}$-NP suspension spectra does not contain the Lorentzian term. The number of DHO profiles most likely to contribute to the measured signal is determined through a Bayesian analysis of measured spectra, as described in detail in Ref.^15^.

## Results and discussion

**Figure 1:**
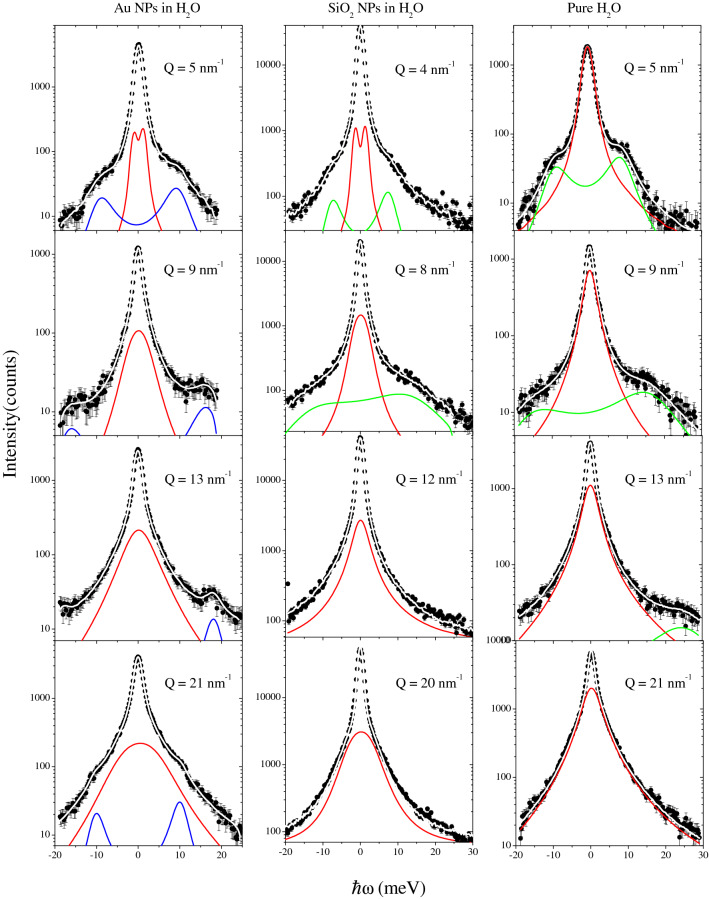
*IXS* spectra (open circles) of the (12 nm negatively charged) $\hbox {vSiO}_2$-NP suspension in water measured at a few representative *Q*’s and presented in this work (middle column) are compared with those we previously measured^24^ on a diluted (0.5 $\%$ weight concentration) aqueous suspension of 15 nm Au-NP (left column) and on pure water (right column). The *IXS* experimental profiles are compared with corresponding best-fit lineshapes (white lines through data) along with their high-frequency (green line) and low-frequency (red line) *DHO* model components (see text). Blue lines in the left column plots are *DHO* contributions arising from phonons in the Au-NP interiors. Notice that, for clarity, elastic and quasi-elastic components of best-fitting lineshapes are not included in the graphs. All samples considered are at ambient conditions.

Spectra collected at a few representative *Q* values are reported in Fig. 1 and compared therein with best-fitting model lineshapes obtained as mentioned in the introductory section and discussed in the “Methods” section in some detail. Plots in the left, middle and right columns of Fig. 1 were collected from a dilute suspension of 15 nm diameter Au-NP in water in a 0.5 $\%$ volume concentration, from an aqueous suspension of 12 nm diameter spherical $\hbox {vSiO}_2$-NP, and in pure water, respectively. In Table 1 the different $\hbox {vSiO}_2$-NP suspensions measured at weight concentrations ranging between 5 and 30$\%$ are listed.

**Figure 2:**
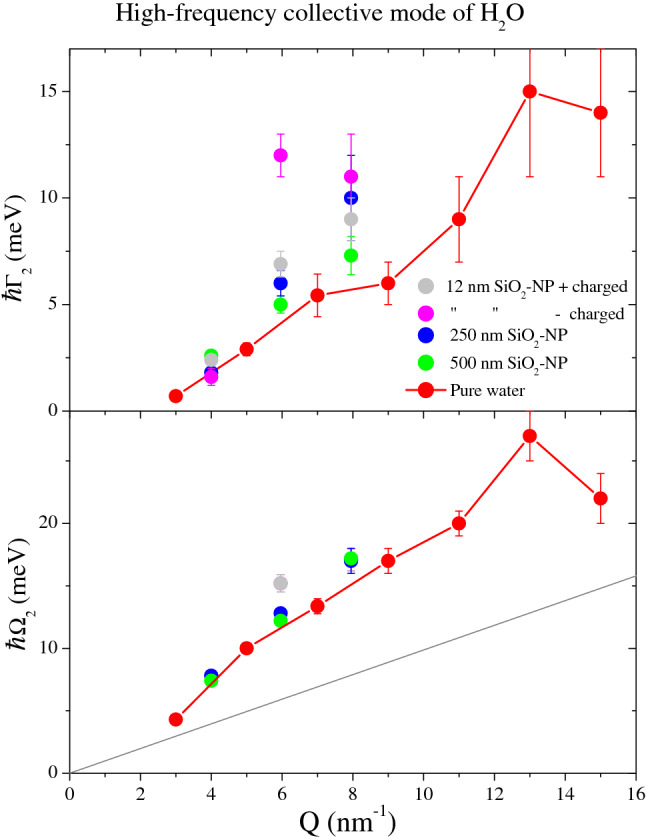
Lower panel: Best-fitting values of the high-frequency Damped Harmonic Oscillator $\Omega _2$ are reported as a function of *Q*. They were obtained from the modeling of *IXS* spectra of aqueous $\hbox {vSiO}_2$-NP suspension and pure water, as indicated in the legend. Previous *IXS* results on pure water from Ref.^4^ are also reported for comparison. The straight line represents the hydrodynamic dispersion law $c_{s}Q$ with $c_s$ being the adiabatic sound velocity of water^30^. Upper panel: corresponding values of the damping coefficient $\Gamma _2$.

**Figure 3:**
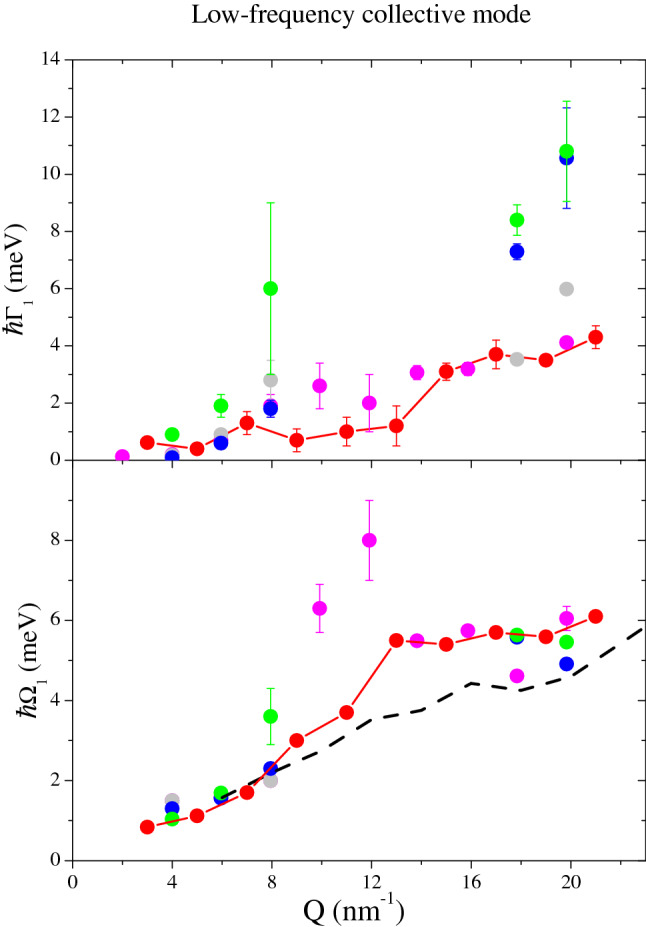
Data are the same as in Fig. 2, but they refer to the low frequency DHO; the dashed line here represents the results of a joint *IXS* and *INS* measurement on heavy water^5^. Symbols as in the legend of Fig. 2.

Measurements in the first and third columns of Fig. 1 were already discussed in Ref.^24^, while the ones in the middle column are the present results. The shape parameters of the best fit curves plotted in Fig. 1 are shown in Figs. 2 and 3 for the high and low excitation modes respectively.

**Table 1:** The $\hbox {vSiO}_2$ nanoparticles in the various aqueous suspensions considered in this work.

Diameter (nm)	Weight concentration	Charge
12	30%	Negative
12	30%	Positive
250	10%	Neutral
500	5%	Neutral

The comparison of various plots prompts some qualitative indications on how dispersed NPs affect the two dominant acoustic modes of water. Such an effect seems substantially different for the high- (green lines) and the low-frequency (red lines) DHO profile, and specifically:

- The high-frequency DHO ($\hbox {DHO}_2$)
  Figure 1 shows that the high-frequency spectral mode of water becomes quite less pronounced when immersed NPs are present; however, as discussed below, in the Au-NP suspension, this mode is hardly visible, the only emerging feature being the Au longitudinal phonon (blue line), while for the $\hbox {SiO}_2$-NP suspension, it persists at least up to 8 $\hbox {nm}^{-1}$ and possibly also at larger *Q* (see also Fig. 6). In pure water, this spectral mode remains instead observable over a wider *Q* range yielding a systematically larger spectral contribution.
- The low-frequency DHO ($\hbox {DHO}_1$)
  Dispersed NPs appear to have a more subtle effect on the low-frequency mode of water. Although this mode is visibly broadened upon NP immersion (see Fig. 3), in all cases, it yields the dominating inelastic contribution to the spectral shape, particularly at high *Q*’s. In pure water, such a mode appears as an unstructured single central peak, well-approximated by an overdamped ($\Omega _1/\Gamma _1< 1$) or critically damped ($\Omega _1/\Gamma _1=1$) DHO component.

Hereafter all shape parameters referring to low-frequency ($\hbox {DHO}_1$) and high-frequency ($\hbox {DHO}_2$) inelastic modes will be labeled by the suffix “1” and “2”, respectively. As mentioned in the introductory section, the Bayesian algorithm privileged a spectral shape model containing either an elastic and a quasielastic component or an elastic component only, which are not reported in Fig. 1. These were approximated by a $\delta$-function (for the elastic contribution) and a Lorentzian profile (for the quasielastic contribution). The former dominates in the suspension spectra, where it can be primarily assigned to the scattering at the liquid-NP interface^18^. Based on the lineshape analysis results illustrated below, we assign low and high-frequency excitations in the $\hbox {vSiO}_2$-NP suspension spectra to the transverse and longitudinal acoustic modes of water^25^, respectively. Notice that the only inelastic feature emerging in the Au-NP suspension spectra (first column in Fig. 1) is substantially sharper than the longitudinal sound mode of water; based also upon this evidence, in our previous work^16^ we assigned this spectral feature to the Au-NP longitudinal phonon, and infer that, for this suspension, the damping of acoustic modes of water is so large to make them disappear from the spectral shape^16,24^. Given the lack of a firm prediction, we can tentatively infer that when a rough match exists between the hypersonic velocities of immersed colloids and hosting liquid, excitations propagating through the latter medium are damped more effectively. In fact, while gold has a high-frequency sound speed similar to the one of water ($\approx$ 3300 m/s)^16,24^, the one of vitreous silica exceeds it by almost a factor two^26^. A test of this hypothesis is definitely worth further experimental scrutiny, to be pursued by joint measurements on colloidal suspensions having components with different sound velocities.

Overall, the persistence of the low-frequency DHO mode in all spectra of Fig. 1 suggests that transverse acoustic propagation can be hindered less easily than its longitudinal counterpart. This inherent “fragility” of the longitudinal mode is somehow consistent with what was observed by Raman scattering measurements, showing that the longitudinal mode’s contribution to the density of states, as opposed to its transverse counterpart, can be easily suppressed upon temperature increase^27^. It is worth recalling that these studies supported the respective assignment of the low and the high-frequency acoustic mode of water to the bending movement of O–O–O triplets belonging to adjacent hydrogen-bonded $\hbox {H}_2\hbox {O}$ molecules and the stretching of O-O pairs projected along the hydrogen bond (HB) direction. The longitudinal and transverse polarization of these acoustic modes is inferred from the comparison with corresponding phonon modes in ice^28^.

Let us now give a closer look at best-fitting parameters extracted from the lineshape modeling of $\hbox {vSiO}_2$ suspensions and pure water spectra.

Best-fitting values of the high-frequency DHO parameters, $\Omega _2$ and $\Gamma _2$, are plotted as a function of *Q* in lower and upper panels of Fig. 2, respectively, and the former curves are compared with previous *IXS* results on water^4^. The $\Omega _2$ values of the suspension are fully consistent with those obtained for pure water, which suggests to assign this dispersive branch to the longitudinal acoustic mode of water thoroughly investigated in the literature^25^. Not unexpectedly, all curves systematically exceed the hydrodynamic linear dispersion $c_{s}Q$ while following, at low-to-intermediate *Q*’s, the characteristic more-than-linear viscoelastic trend revealing the occurrence of a structural relaxation^29^. The consistency between the dispersion curves of pure water and suspensions suggests that viscoelastic properties of bulk water are not drastically affected by the presence $\hbox {vSiO}_2$-NP. Conversely, the upper panel values indicate that the damping $\Gamma _2$ visibly increases upon $\hbox {vSiO}_2$-NP immersion, at least for *Q* larger than 5 $\hbox {nm}^{-1}$. For $Q>8$ $\hbox {nm}^{-1}$ no signature of longitudinal mode appears in the best fitting lineshapes.

We also notice that measured *IXS* spectra bear no evidence for collective modes propagating through the $\hbox {vSiO}_2$-NP interiors, despite the sizable NP concentrations (up 30$\%$ in weight). This circumstance likely owes to the large propagation velocity of the longitudinal acoustic mode of vitreous silica, which, differently from the case of Au, moves its position at or beyond the edge of the spanned energy-window.

Figure 3 displays the shape parameters of the low-frequency DHO ($\hbox {DHO}_1$). Noticeably, data points at intermediate *Q*’s are missing, and the reason will be explained at the end of this section. The $\Omega _1$ values are reported in the bottom graph and therein compared to the transverse acoustic branch of water derived in this work and in joint *IXS* and *INS* measurements on heavy water^5^. The comparison emphasizes a close similarity between the $\Omega _1$ derived from suspension spectra and the one of pure water, customarily assigned to a transverse acoustic mode^4,6^. Concerning the damping coefficient $\Gamma _1$ reported in the upper plot in Fig. 3, it can be readily noticed that its values are consistently higher than their counterparts in pure water, although results at hand do not clarify how this additional damping depends on NP electrostatic charge and concentration while the increase of NP dimension might possibly enhance the *Q*-dependence of the damping.

**Figure 4:**
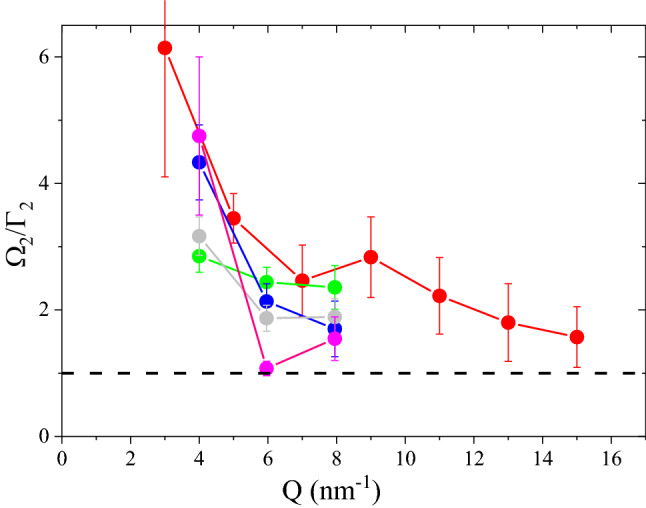
The relative damping $\Omega _{2}/\Gamma _{2}$ obtained using data in Fig. 3 is reported as a function of *Q*. The dashed line shows the critical damping condition. Symbols as in the legend of Fig. 2.

A more quantitative assessment of the relative lifetime of the longitudinal mode can be gained from Fig. 4, where the inverse of the relative damping, that is $\Omega _{2}/\Gamma _{2}$, is reported. It clearly appears that the inclusion of $\hbox {vSiO}_2$-NP in suspensions causes a significant increase of the damping and, consequently, the longitudinal mode propagation in the suspensions becomes overdamped (i.e. $\Omega _{2}/\Gamma _{2}<1$) at *Q* lower than in pure water approaching the critical damping condition. Again, it is unclear to what extent this damping coefficient depends on the characteristics of the considered $\hbox {vSiO}_2$ nanospheres or their relative concentration. Another more general trend of all data is the gradual approach to the critical damping regime upon *Q* increase, which reflects the decreasing ability of the system to support longitudinal (compressional) wave propagation. As demonstrated in Fig. 1, in the high *Q* regime, the low frequency transverse mode yields the overwhelming contribution to the inelastic portion of the spectrum. Considering the mentioned interpretation of acoustic modes of water in terms of intramolecular HB dynamics, it can be recognized that the predominance of transverse excitations at high exchanged wavevectors mirrors the smaller energies required to activate intramolecular HB bending movements as compared to HB stretching ones.

**Figure 5:**
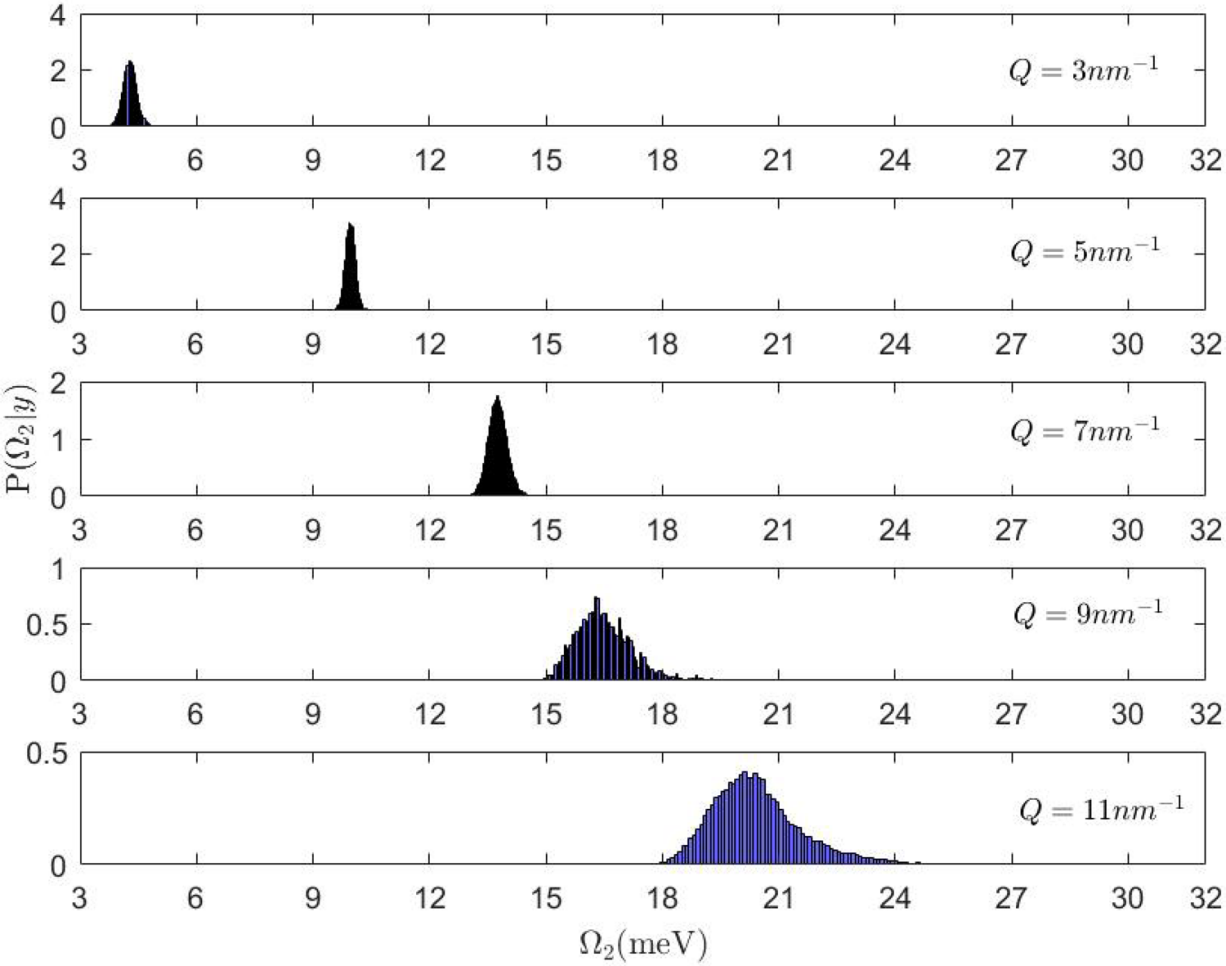
Posterior distribution of the frequency of the longitudinal acoustic mode in pure water, given the available data *y*, at different values of the momentum transfer *Q*.

**Figure 6:**
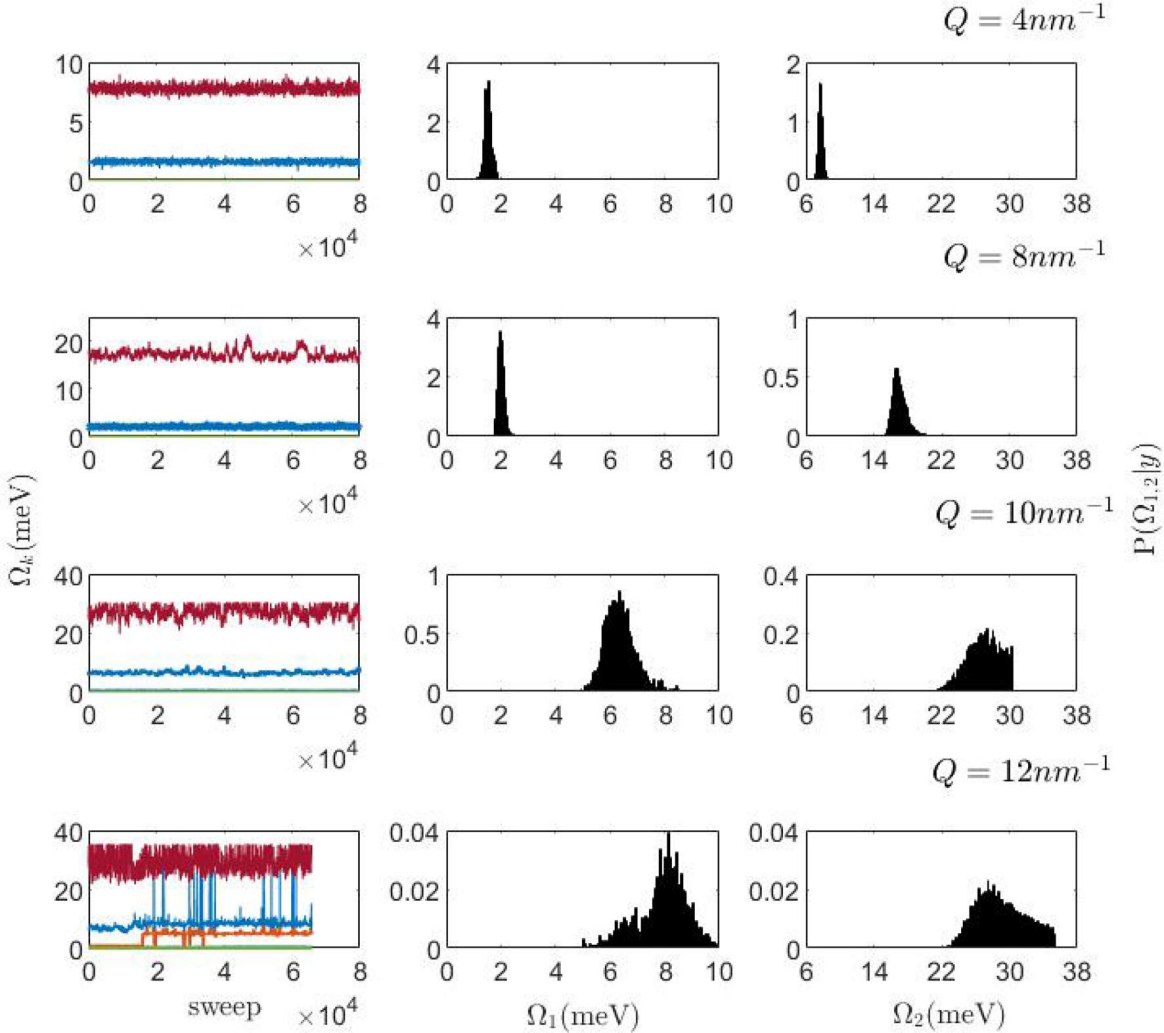
First column: Variation of the $\Omega$ value (traceplot) as a function of the algorithm sweeps at four selected values of the exchanged wavevector *Q*. At the two highest *Q*’s the most probable solution predicts three and four modes respectively. Second and third columns: Posterior distributions conditionally on the measured data *y* for the low (center column) and high (right column) excitation frequencies (see text). To be noticed how the maximum of the posterior density function collapses by orders of magnitude in passing from the case of $\hbox {Q} = 4$ to the case of $Q = 12 \,\hbox {nm}^{-1}$.

The relevant outcome of the Bayesian analysis is summarized in Figs. 5 and 6. In particular, Fig. 5 displays the posterior distribution function of $\Omega _2$ for pure water, as derived in the 3 $\div$ $11 \,\hbox {nm}^{-1}$ *Q*-range from previous *IXS* measurements carried out with the same spectrometer^24^. It appears that the posterior broadens as *Q* increases; this trend owes to the increasing damping and decreasing amplitudes of the $\hbox {DHO}_2$ peak combined with limitations in the spanned energy window, which severely hamper the algorithm’s ability to estimate $\Omega _2$. A comparison with the posterior distributions in the third column of Fig. 6 indicates that the presence of NPs increases even further the uncertainty in the $\Omega _2$ determination when *Q* increases.

As previously mentioned, Fig. 3 lacks some intermediate *Q*’s data; the reason is partly explained by Fig. 6, which summarizes the outcome of the data analysis of the negative charged $\hbox {vSiO}_2$-NP suspension spectra for $Q = 4$, 8, 10, and $12\, \hbox {nm}^{-1}$. It readily appears therein that the posterior distribution of both $\Omega _1$ and $\Omega _2$ rapidly loses sharpness, revealing an increasing uncertainty in the estimate of these parameters. At $Q=10 \,\hbox {nm}^{-1}$, the $\Omega _2$ distribution is abruptly truncated at its maximum, which locates at the edge of the energy window covered by the fit, $E=\hbar \omega _{max}$. For the $Q = 12 \,\hbox {nm}^{-1}$ spectrum, we moved a little further $\hbar \omega _{max}$ which enabled a still reliable inference on $\Omega _2$. However, the unstructured spectral shape at these two *Q*’s deceives the algorithm into privileging an unjustifiably large number of modes ($k = 3$ and 4, for $Q = 10$ and $12\,\hbox {nm}^{-1}$ respectively). For consistency, we refrained from selectively adopting a more constrained prior on *k*^31^ for these *Q*’s only. In summary, the unlikely number of modes identified by the algorithm makes the analysis outcome questionable at intermediate *Q* values. Nonetheless, Fig. 3 includes the values of $\Gamma _1$ and $\Omega _1$ obtained in this *Q*-range for the negatively charged NP suspension, since the posterior of these parameters still remain reasonably well-shaped. We were able to obtain a more consistent outcome at higher *Q*’s, where the transverse mode ($\hbox {DHO}_1$) emerges again from the wings of the central peak.

## Conclusion

We have here discussed the results of Inelastic X-ray Scattering measurements on aqueous suspensions of nanoparticles of different sizes and materials, either neutral or charged. It is worth noticing that, when the dynamics of a disordered system is probed at nanometer scales as in the current *IXS* work, it can no longer be considered isotropic. The resulting anisotropy causes the onset of transverse modes in the spectrum of density fluctuations, whose investigation, in fact, has represented, and still represents, one of the most vivid experimental and numerical research activities in the field of liquid dynamics. A spectral shape modeling based on Bayesian inferential methods enabled us to conclude that the inelastic wings of the terahertz spectrum of these systems are dominated by the two acoustic-like modes of water having either longitudinal or transverse polarization. These inelastic modes have damping systematically enhanced by the presence of nanoparticles in suspension. Consistently with what suggested by previous works^18,24^, we are inclined to ascribe this damping mechanism to the multiple scattering experienced by acoustic modes of water at the NP interface. This multiple scattering enhances mutual interference of these modes, thereby increasing their damping. Interestingly, this damping effect seems substantially more pronounced for golden nanoparticles, due perhaps to the similar high-frequency sound speeds of water and gold. However, we did not find any clear dependence on other parameters of the suspension, such as the nanoparticle’s shape, size, concentration, or electrostatic charge, even if an effect due to the nanoparticles dimension cannot be excluded. We can exclude that the damping effect might be determined by a change in the density of the medium. In fact, a change in the effective (average) density of the medium, in itself, does not explain the observed drastic enhancement of the damping and, even less, the fact that such a damping seems more pronounced in very diluted gold NP suspensions, in which the sparse nature of NPs makes changes in the effective density much smaller. Furthermore, the damping doesn’t seem to depend on concentration (or on the effective medium density) in any systematic way. Overall, we believe that presented results call for a further interpretative effort, as they may inspire new routes in the control and manipulation of terahertz acoustic propagation. In the near future, we expect that additional insight into the observed phenomenon could be gained by studying several systems in the solid phase (below freezing) made out of components with various acoustic matching. Also, a deeper understanding of this effect can be gained by introducing some degree of ordering in the nanoparticle arrangement in the suspension. Finally, we believe that the main result of this genuinely experimental work is to demonstrate the damping effect on the acoustic modes of a liquid induced by dispersed NPs; a more detailed theoretical explanation of this effect would be highly desirable, and possibly these experimental evidences will stimulate a discussion in our scientific community aimed at providing an answer to the puzzling behavior that were highlighted in the current measurement.

## Methods

The measurements have been carried out using the high-resolution *IXS* beamline Sector 30 of the Advanced Photon Source at Argonne National Laboratory^32,33^. The spectrometer was operated with an incident beam energy of 23.7 keV, corresponding to the Si(12 12 12) backscattering reflection from the spherical analyzers. The scattering from the sample was energy-analyzed by 9 independent analyzers mounted on the moving extreme of a spectrometer arm rotating in the horizontal plane and mutually separated by a constant *Q* offset, $\Delta Q$ = 2 $\hbox {nm}^{-1}$. The energy analysis is implemented through the rocking of the crystals of the monochromator unit, while keeping fixed the geometry of the Bragg reflection from the analyzer. The angle between the incident beam and the spectrometer arm and the incident wavelength $\lambda$ determine the *Q* value probed by the nine analyzers. The shape of the energy resolution profile slightly varies for each of the analyzers, with an average spectral full width of about 1.2 meV and a nearly Lorentzian shape. Further details on the spectrometer can be found elsewhere^32^. The negatively charged silica colloids (Ludox LS, 12 nm, 30wt $\%$) and the positively charged silica colloids (Ludox Cl, 12 nm, 30wt $\%$) have been purchased from Sigma-Aldrich company. The neutral silica colloids feature a diameter of 250 nm (10wt $\%$) and 500 nm (5wt $\%$), both purchased from Applied Physics Inc. Si NPs have been chosen for this study because they were thoroughly studied in the past, and, owing to their transparency, also considered in several Brillouin scattering experiments on aqueous suspensions^34–36^. Furthermore, Si, as well as gold NPs have been extensively characterized due to their critical applicative interest. About 30 $\mu$l of each solution is loaded into the capillaries, which are then sealed with wax to allow for *IXS* measurements. The Bayesian inference method used here was first successfully tested on Brillouin neutron scattering data of a crystal of $\hbox {UFe}_2$ already published^37,38^ and successively applied to *INS* measurements on liquid gold, as thoroughly discussed in Ref.^15^, as well as in *IXS*^16^ and neutron Spin Echo^17^ works. We refer the reader to these publications for a detailed description of the used algorithm. We mention here that the model used for the dynamic structure factor consists of the sum of a finite number of components in the form:

1 $$\begin{aligned} S(Q,E)= & {} A_{e}(Q)\delta (E)+[n(E)+1]\frac{E}{k_BT}\Bigg \{ L_{A_0,z_0 }(Q,E)\nonumber \\&+\sum _{j=1}^{k}\frac{2}{\pi }A_{j}(Q)DHO_{j}(Q,E)\Bigg \}\ \end{aligned}$$

where $E=\hbar \omega$ is the energy transferred from the probe particle to the target sample, $\delta (E)$ the Dirac delta function describing the elastic response of the system defined by an intensity factor $A_{e}(Q)$, $n(E)=(e^{E/k_{B}T}-1)^{-1}$ is the Bose thermal factor expressing the detailed balance condition, and the term in curly brackets is the sum of a Lorentzian central contribution—having half-width at half maximum $z_0$ and amplitude $A_0$—accounting for a quasielastic mode - and *k* inelastic contributions, accounted for by Damped Harmonic Oscillator ($DHO_{j}(Q,E)$) terms having amplitudes $A_j(Q)$:

2 $$\begin{aligned} DHO_{j}(Q,E)=\frac{\Omega _{j}^{2}(Q)*\Gamma _{j}(Q)}{(E^2-\Omega _{j}^2(Q) )^2+4[E\Gamma _{j}(Q)]^2} \end{aligned}$$

where $\Omega _{j}(Q)$ and $\Gamma _{j}(Q)$ are the undamped energies and the damping coefficients of the *j*th DHO excitation, respectively. Notice that the number *k* of $DHO_{j}(Q,E)$ excitations likely to appear in the spectrum and their shape coefficients are equally treated as adjustable parameters. To provide an accurate approximation of the measured spectrum, the model function in Eq. (1) must be convoluted with the instrument resolution function *R*(*Q*, *E*) and the result is assumed to sit on the spectral background. Explicitly:

3 $$\begin{aligned} {\tilde{S}}(Q,E)= R(Q,E)\otimes S(Q,E)+B(E) \end{aligned}$$

where *B*(*E*) is a mildly *E*-dependent background intensity.

## References

[1] Maldovan M (2013). Sound and heat revolutions in phononics. Nature.

[2] Deymier PA (2013). Acoustic Metamaterials and Phononic Crystals.

[3] Lu M-H, Feng L, Chen Y-F (2009). Phononic crystals and acoustic metamaterials. Mater. Today.

[4] Pontecorvo E (2005). High-frequency longitudinal and transverse dynamics in water. Phys. Rev. E.

[5] Cunsolo A (2012). Transverse dynamics of water across the melting point: A parallel neutron and x-ray inelastic scattering study. Phys. Rev. B.

[6] Sampoli M, Ruocco G, Sette F (1997). Mixing of longitudinal and transverse dynamics in liquid water. Phys. Rev. Lett..

[7] Bellissima S, Neumann M, Guarini E, Bafile U, Barocchi F (2017). Density of states and dynamical crossover in a dense fluid revealed by exponential mode analysis of the velocity autocorrelation function. Phys. Rev. E.

[8] Bellissima S (2016). The hydrogen-bond collective dynamics in liquid methanol. Sci. Rep..

[9] Guarini E (2017). Density of states from mode expansion of the self-dynamic structure factor of a liquid metal. Phys. Rev. E.

[10] Guarini E, Neumann M, Bellissima S, Colognesi D, Bafile U (2019). Density dependence of the dynamical processes governing the velocity autocorrelation function of a quantum fluid. Phys. Rev. E.

[11] Guarini E (2020). A neutron brillouin scattering and ab initio simulation study of the collective dynamics of liquid silver. Phys. Rev. B.

[12] Giordano VM, Monaco G (2010). Fingerprints of order and disorder on the high-frequency dynamics of liquids. Proc. Natl. Acad. Sci..

[13] Giordano VM, Monaco G (2011). Inelastic x-ray scattering study of liquid Ga: Implications for the short-range order. Phys. Rev. B.

[14] Bolmatov D (2017). Emergent optical phononic modes upon nanoscale mesogenic phase transitions. Nano Lett..

[15] De Francesco A, Guarini E, Bafile U, Formisano F, Scaccia L (2016). Bayesian approach to the analysis of neutron Brillouin scattering data on liquid metals. Phys. Rev. E.

[16] De Francesco A (2018). Damping off terahertz sound modes of a liquid upon immersion of nanoparticles. ACS Nano.

[17] De Francesco A (2019). Model-free description of polymer-coated gold nanoparticle dynamics in aqueous solutions obtained by Bayesian analysis of neutron spin echo data. Phys. Rev. E.

[18] De Francesco A (2020). Onset of interfacial waves in the terahertz spectrum of a nanoparticle suspension. Phys. Rev. E.

[19] De Francesco A (2020). Shaping the terahertz sound propagation in water under highly directional confinement. Phys. Rev. B.

[20] Sinha SK (2001). Theory of inelastic x-ray scattering from condensed matter. J. Phys. Condens. Matter.

[21] Squires GL (1996). Introduction to the Theory of Thermal Neutron Scattering.

[22] Gelman A (2013). Bayesian Data Analysis.

[23] Sivia D, Skilling J (2006). Data Analysis: A Bayesian Tutorial.

[24] De Francesco A (2020). The terahertz dynamics of an aqueous nanoparticle suspension: An inelastic x-ray scattering study. Nanomaterials.

[25] Cunsolo, A. The THz spectrum of density fluctuations of water: The viscoelastic regime. *Adv. Condens. Matter Phys.* **2015** (2015).

[26] Baldi G, Giordano VM, Monaco G, Ruta B (2010). Sound attenuation at terahertz frequencies and the boson peak of vitreous silica. Phys. Rev. Lett..

[27] Walrafen G (1964). Raman spectral studies of water structure. J. Chem. Phys..

[28] Petrenko VF, Whitworth RW (1999). Physics of Ice.

[29] Monaco G, Cunsolo A, Ruocco G, Sette F (1999). Viscoelastic behavior of water in the terahertz-frequency range: an inelastic x-ray scattering study. Phys. Rev. E.

[30] Kestin J, Sengers J, Kamgar-Parsi B, Sengers JL (1984). Thermophysical properties of fluid H$$_2$$O. J. Phys. Chem. Ref. Data.

[31] De Francesco A, Cunsolo A, Scaccia L, Cunsolo A, Franco MKKD, Yokaichiya F (2020). Bayesian approach for x-ray and neutron scattering spectroscopy. Inelastic X-Ray Scattering and X-Ray Powder Diffraction Applications, Chap. 2, 26.

[32] Said AH, Sinn H, Divan R (2011). New developments in fabrication of high-energy-resolution analyzers for inelastic x-ray spectroscopy. J. Synchrotron Radiat..

[33] Toellner T, Alatas A, Said A (2011). Six-reflection meV-monochromator for synchrotron radiation. J. Synchrotron Radiat..

[34] Liu J, Ye L, Weitz D, Sheng P (1990). Novel acoustic excitations in suspensions of hard-sphere colloids. Phys. Rev. Lett..

[35] Penciu R, Fytas G, Economou E, Steffen W, Yannopoulos S (2000). Acoustic excitations in suspensions of soft colloids. Phys. Rev. Lett..

[36] Penciu R, Kriegs H, Petekidis G, Fytas G, Economou E (2003). Phonons in colloidal systems. J. Chem. Phys..

[37] Paolasini, L., Formisano, F., Caciuffo, R., Lander, G. & Lapertot, G. Giant magnetoelastic interaction in UF$$\text{e}_2$$. I*J. Phys. Conf. Ser.* **340**, 012063 (IOP Publishing, 2012).

[38] De Francesco, A. & Scaccia, L. Bayesian model selection and parameter estimation of inelastic neutron scattering spectra. https://cdn.eventsforce.net/files/ef-q5vmtsq56tk6/website/192/complete_abstract_book.pdf (2013).

